# Dementia and COVID-19 among Older African American Adults: A Scoping Review of Healthcare Access and Resources

**DOI:** 10.3390/ijerph20043494

**Published:** 2023-02-16

**Authors:** Idorenyin Imoh Udoh, Elias Mpofu, Gayle Prybutok

**Affiliations:** 1 Rehabilitation and Health Services, University of North Texas, Chilton Hall, 410 Avenue C, Suite 289, Denton, TX 76201, USA; 2 School of Health Sciences, University of Sydney, Camperdown, NSW 2050, Australia; 3 Educational Psychology, University of Johannesburg, Johannesburg 2000, South Africa

**Keywords:** healthcare accessibility, resources, dementia, COVID-19, older adults, African Americans, healthcare quality, structural inequity, health inequalities

## Abstract

African American/Black communities comprise 12.2% of the U.S. population, with a COVID-19 infection rate of more than 18% and marginal access to healthcare services. This scoping review synthesizes the emerging evidence on healthcare accessibility among older African American adult communities with dementia and COVID-19, as well as the resource requirements for this population during the pandemic. Searches of different databases for empirical studies and other sources on dementia and COVID-19 among older African American adults yielded 13 studies that met the following inclusion criteria: (a) focus on dementia and COVID-19, (b) sampled older African American adults, (c) investigated healthcare accessibility and resources, and (d) published between 2019 and 2022. Following the initial selection of the studies, eight were selected for relevance based on the Population, Concept, and Context (PCC) inclusion and exclusion criteria. Thematic analysis indicated that older African Americans with dementia and COVID-19 experienced longer delays in accessing timely healthcare, including transportation, intensive care units (ICUs), and mechanical ventilation. They also had reduced healthcare resources associated with a lack of health insurance, low financial resources, and an increased length of hospital stay, which further aggravated the negative effects of comorbid dementia and COVID-19 infections. Evidence showed that racial and age disparities affected older African American adults with dementia and COVID-19, resulting in lower healthcare access and marginal resources. This is consistent with historical and systemic inequities in meeting the healthcare needs of people of color in the United States, which was compounded for older African Americans during the COVID-19 pandemic.

## 1. Introduction

Dementia is a clinical ailment in older adults characterized by a widespread decline in cognitive abilities, which affects normal daily functioning [1,2]. African Americans make up approximately 9% of the U.S. older adult population or an estimated 5 million people, which is expected to increase to about 12 million by 2060 [3]. The majority of older African American adults have low incomes and are less able to afford health insurance [2]. About 10.7% of adults aged 65 years and over in the United States have dementia [4,5]. Dementia disproportionately affects African Americans and Hispanics compared to Caucasians, with a prevalence that is twice the national average [6]. Moreover, the risk of mortality from COVID-19 is higher among African Americans at about 2.5 to 3 times higher than among White or Hispanic groups [7]. The fact that older adults are highly vulnerable to contracting COVID-19 creates a double jeopardy for individuals with dementia [8]. COVID-19 is a respiratory disease transmitted by airborne droplets of saliva and discharge from the nose when an infected person sneezes or coughs [9].

Racial minorities in the U.S. may be less likely to be diagnosed with dementia due to poorer access to healthcare [7]. Patients with dementia may not understand quarantine measures, thereby exposing themselves to a higher likelihood of infection and poorer follow-up with treatment if infected [5,10]. These treatment adherence limitations could add to the burden on them and their service providers and increase the risk of infecting others. Furthermore, the fact that many older adults of racial minorities may be living in neighborhoods with fewer resources and long-term care facilities [9] could increase their risk of infection from COVID-19 due to greater exposure within the community. Although the U.S. healthcare system is technologically advanced, the health outcomes for older African American adults are below par due to racial disparities, structural inequities, and difficulties navigating the healthcare system [8,9]. This scoping review aggregates the evidence on health access disparities and resources for older African American adults with dementia and COVID-19.

### 1.1. Disparities in Healthcare Affecting Older African American Adults

Emerging studies have shown that minority groups in the U.S. are disproportionately affected by COVID-19 and experience unequal distribution of health resources [11,12,13]. For example, a study reported that in the state of Michigan, 41% of people who have died from COVID-19 were black or African American compared to a 28% death rate for Caucasians [14]. Similarly, in the state of Louisiana, African Americans make up about 58% of COVID-19-related deaths [13]. In New York City, Hispanics and African Americans make up 29.7% and 30.5% of COVID-19-related deaths, respectively [11]. Regrettably, older African American adults may experience healthcare disparities associated with inadequate health insurance [15]. They may be overly reliant on emergency room visits rather than visiting the same doctor regularly [16]. These healthcare access disparities are compounded by comorbid dementia and COVID-19, leading to deterioration to the advanced stages of the ailments and risking premature mortality [17]. Further investigation is needed to understand how health resources and access to care among older African American adults with dementia and COVID-19, as a racial minority, may be associated with their health outcomes.

### 1.2. Healthcare Accessibility and Resources

Healthcare accessibility refers to the ease of availability of appropriate services for the best possible health outcomes [18]. This is defined by the approachability, availability, and appropriateness of the provider in terms of a patient’s needs. Healthcare access refers to a patient’s sense of acceptance by the provider [18]. Reduced healthcare access has been a primary cause of health disparities among older African American adults, most of whom are on Medicaid, a U.S. government health insurance program for vulnerable populations including the indigent elderly. Nonetheless, Medicaid has limitations that restrict access to elective treatments and payment for proven, brand medicines and devices that would benefit low-income older adults [19]. Differences in access, use, and quality of care can measurably determine health and healthcare outcomes for people and older adults living with dementia and COVID-19 [20]. These outcomes may include financial pressure for patients and their families, premature nursing home admissions for people with dementia, and caregiver well-being.

Access to preventive and care services for older African American adults living with dementia and COVID-19 presents opportunities for improved quality of care within their neighborhoods, including nursing homes and assisted living facilities [21]. However, at the individual level, systemic factors, such as a lack of insurance, poor financial status, risks, and discrimination, may be obstacles to accessing quality care [22] for older African American adults, who are also more likely to not have insurance [23]. Similarly, at the healthcare system level, organizational inefficiencies, service abilities, and racialized ageism affecting the attitudes and perceptions of healthcare professionals toward older African American adults may diminish healthcare accessibility [23,24,25]. Access to services for older African Americans could be worsened due to their more significant residential mobility issues, reduced cell phone access, and transportation limitations [26,27].

Health resources refer to the various available means that ensure well-being, including financial stability, education, transportation, food security, housing, information, and communication aids [28,29]. For older adults with dementia and COVID-19, a lack of resources may be catastrophic [29], exacerbating their already high vulnerability [30].

### 1.3. Current Study

We sought to explore the emerging evidence on healthcare accessibility and health resources issues related to older African American adults with dementia and COVID-19. For this, we aimed to address the following research questions:

1. What is the emerging evidence on healthcare accessibility for older African American adults with dementia and COVID-19?

2. What are the healthcare resource issues affecting older African American adults with dementia and COVID-19?

## 2. Methods

### 2.1. Research Design

A scoping review identifies gaps in the emerging body of evidence [31,32,33] and, in the case of dementia and COVID-19, more focused studies on aspects of evidence-informed healthcare support interventions. Moreover, scoping reviews are mainly conducted to deliver an overview of the emerging evidence by study type and methodological quality or risk of bias since the studies on emerging health conditions are exploratory and the data are preliminary [34]. Therefore, a scoping review was appropriate for this study to aggregate and profile the emerging research evidence on healthcare accessibility and resource disparity issues for older African American adults with dementia and COVID-19.

### 2.2. Search Strategy

We searched Ebscohost, Medline, Academic search complete, Ageline, the Atla Religion Database with AtlaSerials, PsycInfo, and the Psychology and Behavioral Science Collection using the terms ‘healthcare accessibility’, ‘resources’, ‘African American older adults’, ‘dementia’, and ‘COVID-19’ (see Table 1). We collaborated with our research librarian, who recommended using Medline and PubMed to retrieve peer-reviewed resources in the field of medicine and health focused on dementia, COVID-19, healthcare accessibility, resources, and African Americans. The search was undertaken between June 2021 and September 2022. After this, we expanded our search terms to include those shown in Table 1.

### 2.3. Inclusion/Exclusion Criteria

This stage involved selecting the articles in three steps: (1) title screening, (2) abstract screening, and (3) full article screening. As per the inclusion and exclusion criteria, studies were selected based on the Population, Concept, and Context (PCC) framework [32]. Studies were selected if they included (1) older African Americans or older Black adults, (2) dementia, (3) COVID-19, (4) healthcare accessibility, (5) health resources, and (6) disparities (see Table 2). The included studies were published from December 2019 to September 2022. The search terms for dementia also included Alzheimer’s and cognitive decline, and articles that discussed cognitive impairment were part of the inclusion criteria for our search. The search terms for COVID-19 included coronavirus and SARS-CoV-2. Studies were excluded if they were published in a language other than English.

### 2.4. Data Screening

The lead author performed the literature search, which was agreed to by the second author. We extracted essential data qualities based on the study design (i.e., cross-sectional, longitudinal, and empirical), participant characteristics (i.e., older African American adults with dementia, older African American adults with COVID-19), and outcomes (healthcare accessibility and resources issues). The third author assisted in considering the relevance to the topic and probable links to the inclusion criteria, and emerging disagreements were resolved by consensus. 

### 2.5. Summary of Literature Search

**Figure 1:**
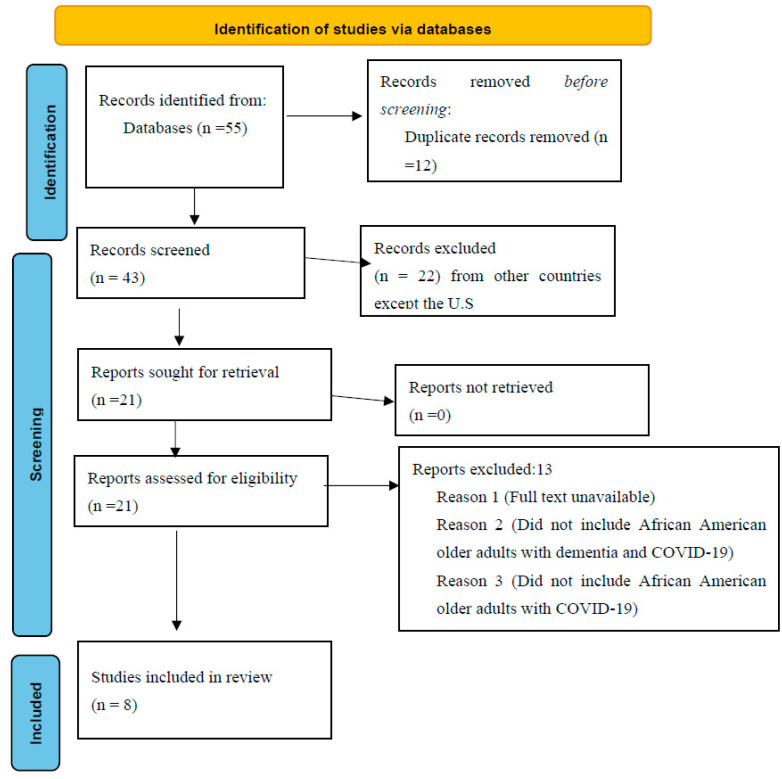
Flowchart showing the number of publications identified and screened for eligibility during the scoping review.

Figure 1 presents a flowchart of the selection process of the studies. The initial database search yielded 55 pertinent studies, of which 12 were duplicates. Further screening of the remaining 43 studies by the lead author led to the exclusion of 22 publications due to marginal relevance and the retention of 21 documents for additional screening. This process led to the rejection of thirteen studies due to inclusion criteria issues. The selection process outcome yielded 8 studies for the present scoping review by consensus of the authors. The low number of studies meeting the inclusion criteria was expected, as COVID-19 is a new community-spread virus and has attracted healthcare interest only in the past 2–3 years (or since its global spread from December 2019 onward).

### 2.6. Data Organization

We organized the data from the articles by author/year, study design, health sustenance outcomes (healthcare accessibility and resources), COVID-19/dementia, and participants’ characteristics. We considered the dementia and COVID diagnoses in each study and included a separate column to ensure our focus on these factors.

## 3. Results

### 3.1. Data Synthesis

This study followed the procedures of Aveyard for synthesizing evidence [41] and was framed by Levesque’s conceptualization [42]. The Aveyard procedure involves becoming familiar with the evidence, generating initial themes, reviewing the articles to determine if they relate to healthcare accessibility and the resource indicators specify the resources, and drawing final themes by consensus among the authors. Levesque’s patient-centered healthcare conceptual models allow for a comprehensive assessment of the multi-layered processes of access both in the healthcare system and population contexts, suggesting potential room for improvement. Access to healthcare is described as the opportunity to identify, seek and receive healthcare to ensure that the needs of patients are met [42].

All authors then discussed the themes resulting from the analysis by continuously relating them to the research questions of this study guided by Levesque’s conceptual framework.

### 3.2. Findings

The majority of the studies (n = 5, 87.5%) used in this review reported on healthcare accessibility and we reported our results based on Levesque’s healthcare access framework on acceptance, approachability, availability, and appropriateness (26,27,35,36,37,38,39) (see Table 3). Although the authors did not specifically analyze Levesque’s framework, they nevertheless utilized terms similar or identical to those in the framework. Half of the studies reported the availability (or lack) of healthcare resources due to financial insecurities, a lack of healthcare insurance, education/knowledge gaps, transportation, Internet/phone or telemedicine, cognitive screening, ambulatory care, and so on ([26,36,37,38,39]) (See Table 4). These were grouped as materials, financial insecurities, and knowledge gaps. Four studies (50%) focused on a combination of accessibility and resourcing associated with insurance and Internet/telemedicine utilization ([36,37,38,39]). We discuss each of the emerging themes and subthemes below (See Table 3 and Table 4).

### 3.3. Participants’ Characteristics

The participants of interest in this review were older African American adults (60 to 75 years) with dementia and COVID-19. The sample size of the participants used in this study ranged from N = 16 to N = 53,640,888. One study focused on dementia and altered mental status among older African American adults with dementia, noting the presence of comorbidities such as kidney and cerebrovascular disease [26]. One study noted the absolute lack of insurance of participants [38] In addition, two studies reported that participants had Medicaid [39] and Medicare [36], although they had issues with accessing associated benefits. Participants in the remaining six studies used private insurance. Finally, one study included participants’ caregivers [39]. Seven studies included participants from all ethnic groups. Only one study reported all participants as older African American adults [35]. The social determinants of health for the participants in the studies included low income, living in poor communities where transportation was generally a source of concern, and an over-reliance on public health facilities [36,37,39] (See Table 2). 

### 3.4. Healthcare Accessibility Disparities

Our findings suggest vulnerabilities impacting older African American adults with dementia and COVID-19 regarding their access to resources for healthcare services [36,39]. We measured healthcare accessibility based on geographic impediments, the capacity of healthcare, and demand in the affected population [36] (see Table 3). We considered the categories outlined below. 

### 3.5. Acceptance by Healthcare Systems

The findings of the included studies showed many healthcare access disparities for older African American adults with dementia and COVID-19 due to acceptance issues by healthcare professionals [35,36,38] (See Table 3). Differences existed in care acceptance between older African American adults with dementia and COVID-19 and those with Medicare and private insurance. One study aimed to identify differences in feelings of loneliness, sadness, and social disconnection early in the pandemic across racial groups and the possible mitigating factors [36]. In this study with 8125 beneficiaries, older African American adults were least likely to report feeling socially disconnected (odds ratio = 0.55; CI: 0.42–0.73; *p* < 0.001). However, Internet access was associated with increased odds of social connectedness in older African American adults [36]. Nonetheless, community-dwelling older adults with dementia and COVID-19 on Medicaid reported disparities in access to healthcare facilities [38], suggesting lower connectedness to social networks for health and well-being. 

Although the transition of outpatient visits to telehealth platforms may have increased care acceptance, older African Americans were not likely to engage effectively in telemedicine without the necessary help [36]. In addition, ineffective outpatient care and reduced inpatient admission rates increased mortality and lowered the quality of life among older African American adults with dementia and COVID-19 [36,38]. The risks of lowered care acceptance by older African Americans may be compounded by their perception of a lack of person-centered and culturally sensitive care for them [26]. Moreover, individual experiences of care, such as knowledge of services and their accessibility, could strengthen provider–patient relationships. Finally, acceptance issues were compounded by prevalent pre-existing conditions, and multiple conditions increased the incidence of severe complications from COVID-19 among older African American adults [36].

**Approachability**: Approachability refers to the ability to approach healthcare facilities or professionals [43]. Approaching healthcare facilities and professionals presumes being listened to and valued in the treatment process. Studies suggest systemic and structural disparities are issues related to healthcare accessibility for older African American adults with dementia and COVID-19 [35,36,39]. As an example, most older African American adults referred to in the studies lived in rural communities or densely packed locations, where access to timely and proper transportation they could use to access quality care was lacking [36,39]. Furthermore, one study noted a disproportionate under-representation in the number of Black people with dementia and COVID-19 in healthcare records, suggesting a limited approach to healthcare facilities and professionals in Black neighborhoods [39]. In a study that aimed to explore the impact of COVID-19 on Black and South-Asian older adults living with dementia and their carers, as well as explore the experiences of dementia care, older African American adults living with dementia were more likely to be diagnosed later and have less access to healthcare and social support than their White counterparts [36]. 

COVID-19 has exacerbated health inequalities and diminished trust in government and health services in underserved communities. People living with dementia need support at all levels and this study highlighted how the pandemic has impacted each level. Ways to improve trust in the government and health professionals, alongside culturally adapted health messaging, should be explored. Additionally, an examination of how cultural values and norms can influence help-seeking responses to dementia and increase trust in services may be helpful post-pandemic. This study revealed that the pandemic impacted people from ethnic minority backgrounds living with dementia and their carers due to their low socioeconomic status. A lack of trust in the government and increased anxiety due to the media were reported. There was a perceived lack of person-centered and culturally sensitive care from healthcare professionals and concerns around care homes as places of safety. Participants’ relationships with their families and communities, their knowledge of services, and their identities and faiths influenced their experiences during the pandemic [36].

Disparities in approachability in terms of healthcare accessibility led to longer waiting times for older African Americans with dementia and COVID-19 seeking care [38]. A study noted the challenges associated with mobility and reduced cell phone access due to a lack of amenities for healthcare follow-ups for older African Americans with dementia and COVID-19 compared to their White and Hispanic counterparts [36]. Other studies showed a general lack of trust by older African American adults in healthcare professionals and the government due to discrimination and fear [26,38,39]. Other studies reported poor medical interactions [38] and a lack of access to technology [27], thereby inducing social isolation among people with dementia and COVID-19.

**Availability**: Availability of services refers to the ability to obtain the right care at the right time [18,44]. Older African American adults with dementia and COVID-19 experienced care delays and difficulties in securing healthcare appointments, which may have worsened their condition and increased their risk of mortality [26]. In another study, a large percentage of older African American adults with dementia and COVID-19 could not access healthcare due to significant memory issues, as they could not remember their healthcare provider and were also not contacted [39]. Another study showed that remote engagement was possible for recruiting, educating, and conducting cognitive screening with rural older adults during a pandemic [39]. This implied that older African American adults needed educational resources to improve their knowledge of dementia and strategies for sustaining a good quality of life. 

Additionally, studies reported the inability of older African American adults to secure appointments and connect with family and healthcare professionals due to a lack of access to the Internet [36,39] and care delays reduced successful healthcare delivery to older African American adults compared to their White counterparts [27]. Older African American adults reported an inability to access quality care, participate in medical appointments, or communicate with their loved ones. As a result, they were more likely to terminate the care support services they were receiving out of frustration [38]. The caregivers of older adults with dementia and COVID-19 had insecurities about the virus and indirect pandemic effects, such as their loved ones and care recipients experiencing confusion, loneliness, and poor care [36,38].

**Appropriateness**: This refers to the quality of healthcare access and whether it is suitable or proper [18] in the case of older African American adults with dementia and COVID-19. Cultural values and beliefs regarding dementia and the care of older people differ. Therefore, there is a need for cultural sensitivity in the provision of person-centered dementia care for older African American adults [45]. The absence of close observation after hospitalization and early intervention after diagnosis was prevalent among older African American adults [26,38]. 

Structural inequities, such as lower ICU admission and mechanical ventilation, were also predominant among this ethnic group or were not administered when needed [26]. A greater risk of COVID-19 infection among older African American adults was associated with other sociodemographic and environmental characteristics, such as location and educational levels, which negatively impacted the level, type, and appropriateness of care and resources accessed [24,37]. Additionally, older African American adults were more likely to live in communities with poor air quality, work in jobs that affected their mobility and impeded access, and lack access to healthcare through insurance and funds to pay for the appropriate care. This likely led to increased risks of infection, prolonged diagnoses, and racial disparities in mortality, especially in nursing homes [27].

### 3.6. Healthcare Resource Disparities

The reviewed studies reported healthcare resources based on the availability of funds, materials, personnel, facilities, knowledge gaps, and other items that can be used to ensure the quality of care for older African American adults with dementia and COVID-19. We elaborate on this theme and its sub-themes below (See Table 4). 

**Financial insecurities and lack of health insurance**: This involves not having enough funds and resources to meet healthcare needs [26,38]. Older African American adults with dementia and COVID-19 had poor or low finances and could not access the needed or best resources to take care of their health. Two studies reported that due to financial insecurities, older African American adults lacked the proper insurance to take care of their health needs [36,39]. Specifically, a lack of insurance due to limited income and poor financial status led to issues with accessing the appropriate health resources [36]. This meant that they were unable to afford the proper care and medication for their particular situations, leading to high mortality among older African American adults with dementia and COVID-19 [37,38]. Despite having Medicare, older African American adults with dementia and COVID-19 still experienced difficulties accessing the healthcare services they required [38]. As a result of financial and health insurance challenges, managing dementia and COVID-19 comorbidities was complicated for those of low socioeconomic status or with limited incomes. For example, in a retrospective study of patients admitted to an urban safety net hospital in New Orleans, Louisiana, with reactive COVID-19 and the presence of dementia, older Black patients showed longer symptom duration at presentation than their White counterparts (6.41 vs. 5.88 days; *p* = 0.05) [38].

**Internet, telephone, and transportation service constraints:** Older African Americans lacked access to the Internet and telephone services required for timely access to health services for those with dementia and COVID-19 [36], particularly those in rural areas [39]. This is despite the fact that telephone-based cognitive screening intervention could be effective in increasing dementia knowledge, detecting the need for further cognitive evaluation, and creating and tracking the results of referrals [36]. There was also a lack of Internet and phone access to connect to support groups, family, and peers [36]. Most importantly, these communication challenges extended to the family caregivers of older African Americans with dementia and COVID-19, who were unable to access long-term care facilities due to COVID-19 restriction policies [38]. Regardless, many caregivers of people with dementia reported that the availability of telemedicine and other remote healthcare interventions improved care services for older patients [38]. Some studies reported a lack of transportation to address participants’ healthcare needs [38,39].

### 3.7. Materials and Support Resources

**Social support resources**: The lack of proper ongoing social support led to poor outcomes in the quality of care among older African American adults with dementia and COVID-19 [26,36,37]. In addition, older African Americans with dementia and COVID-19 were less likely to experience mechanical ventilation and ambulatory care [26,37]. Compared to other racial/ethnic groups, older African Americans lacked social relationships and were at higher risk of loneliness, sadness, and social disconnection during the pandemic [35]. Additionally, older African American adults reported dissatisfaction and feelings of social disconnection [36]. Compared to Asians and other minority ethnic background populations, the care experiences of older African Americans with dementia were noted as being more disruptive to their daily lives [36] (See Table 4).

**Mechanical ventilation and ambulatory care resources**: Compared to other ethnic groups, older African American adults with dementia had low access to mechanical ventilation, a higher need for intensive care unit (ICU) care and dialysis, and an increased length of stay [26]. Older African American adults with dementia had a history of a lack of ambulatory care, which led to significantly higher rates of hospitalization and more severe outcomes [37].

**Knowledge gaps**: Many older African American adults with dementia had limited knowledge of cognitive screening [37]. One study discussed participants’ lack of knowledge of dementia, COVID-19, and how to care for themselves and avoid infecting others. Some older African Americans with dementia and COVID-19 desired to avoid future nursing home care and were hesitant about seeking healthcare until the pandemic had passed [36,38]. The fact that the majority of older African American participants had little or no education beyond a high school degree further exacerbated the structural inequities in their knowledge of self-managing dementia and COVID-19 [38]. Additionally, older populations who can be described as vulnerable individuals with low socioeconomic status were not likely to have adequate information about electronic health records to assess their ambulatory history for ambulatory care [37].

### 3.8. Discussion and Implications

This scoping review explored the emerging evidence on the disparities in healthcare accessibility and availability of resources among older African American adults with dementia and COVID-19. Our findings are consistent with the extant findings on racialized health disparities in the United States, which can be extended to the COVID-19 pandemic experience. Historically and contemporarily, older African Americans have lower acceptance rates for care [4,5,36,38,44,46]. They also may find healthcare facilities less approachable and trustworthy [25,33,36,38,44,45], further limiting healthcare service usage during a pandemic due to a lack of resources [29,36,39,44,46]. This suggests that prevention efforts should include strategies and knowledge through the dissemination of appropriate information to limit SARS-CoV-2 exposure and transmission in Black communities, especially in older black adults, as a step toward reducing COVID-19-related racial inequities [38].

The population of the United States of America is vastly diverse and due to their intersectionality, different groups may have vastly discrepant healthcare system experiences. As part of the American culture [47], older African Americans have experienced some form of disparity in healthcare accessibility and healthcare resources because of differences in ethnicity, age, and segregation. This has resulted in differing standards, opinions, and differences in value systems. There are perceptions among African Americans that healthcare professionals intentionally treat racially disadvantaged and vulnerable people differently from their Caucasian counterparts [48]. These perceptions tend to increase with age and are much worse in older age groups, as older people have more healthcare vulnerabilities from the aging process itself. Conditions such as dementia and a community-spread pandemic only exacerbate healthcare disparities affecting older African Americans.

A vast number of older African Americans are underinsured or not insured [6,8]. The lack of access directly reflects the quality of healthcare received by older African Americans and other minorities. African Americans are more likely to require healthcare services but are unlikely to receive appropriate care. This absence of quality care and access to care creates a mistrust of the system. Recent studies [11,18,48] have shown that even when older African Americans access healthcare systems and have the means to pay for services, they are unlikely to receive the same level of care as their Caucasian counterparts. A critical concern for older African Americans is that many of them access medical care through large public health systems and resources for care, such as medical ventilation, intensive care units, and healthcare information, are severely limited and, in most cases, reserved for their Caucasian counterparts. These historic race-based vulnerabilities affecting African Americans persist in older age groups and become worse with age-related cognitive decline in the presence of COVID-19 [11,12,14,46].

Poverty as a result of low income has been identified as a primary indicator of the absence of basic human needs such as food, clothing, healthcare, education, and shelter [48]. Older African Americans are among the poorest ethnic group in the United States, and this has been highly correlated with adverse health outcomes and an increase in morbidity and mortality [22,24,25]. Older African Americans live in communities with the lowest housing quality, predisposing them to communicable community-spread diseases such as COVID-19 [12,46]. Those with dementia living in the poorest neighborhoods would have less access to quality healthcare and treatment resources. The lack of transportation is also an issue in low-income African American communities and is a vital barrier to accessing quality healthcare services, especially preventive healthcare.

Furthermore, chronic illnesses, including diabetes, cardiovascular diseases, obesity, heightened blood pressure, and increased blood lead levels, are prevalent among individuals with low socioeconomic status. These chronic illnesses represent vulnerabilities for COVID-19 infection and compound aging with or without dementia.

These findings suggest the need for targeted interventions for addressing known health disparities due to care inaccessibility or availability during the COVID pandemic, and new variants for which vaccines have not yet been developed appear to spread rapidly. Depending on access to healthcare services and health resources, older African American adults with dementia and COVID-19 may experience avoidable increased mortality due to the poor quality of care. Additionally, the evidence that Medicare subscription by older African American adults with dementia and COVID-19 did not alleviate healthcare access and resources issues [36,49] shows that although having health insurance is important, it is not sufficient on its own to affect access to healthcare and resources. Rather, there is a need for structural change in the United States healthcare system to be more inclusive of care needs across the racial divide to reduce vulnerabilities during pandemics, which may be the new norm in our globalized world community.

**Table 3:** Summary of evidence of issues with healthcare accessibility among older African American adults with dementia and COVID-19.

##	Components	Attributes	Studies
1	Approachability	Systemic and structural disparities, transportation, disproportionate representation, healthcare professional attitudes, poor medical interaction	[26,27,37,38,39,40]
2	Acceptability	Medicare and private insurance, comorbidities, age, ethnicity, appointment availability, and limited healthcare facilities.	[26,35,36,38,39]
3	Appropriateness	Physician suitability, timely care, appropriate care.	[26,27,37,39]
4	Availability	Appointments, healthcare professionals, facilities, and lack of digital communication with patients. Low ratio of professionals to patients, poor access to technology	[26,27,36,38,40]

**Table 4:** Summary of evidence of resources to mitigate adverse outcomes in older African American adults with dementia and COVID-19.

#	Components	Attributes	Studies
1	Financial insecurities	Low income, lack of insurance, and insurance issues.	[36,38,39,40]
2	Knowledge gaps	Lack of appropriate knowledge about dementia, COVID-19, and how to care for themselves to avoid exaggerating their condition. Lack of protective strategies and appropriate information.	[37,39]
3	Materials, personnel, facilities, funds	Low income, poor socioeconomic status, lower education, lack of informational materials, limited health facilities, limited facilities such as ICU, mechanical ventilation, absence of oxygen requirements, structural inequities, and inadequate support structures such as cognitive screening.	[26,36,37,38]
4	Internet, telephone, transportation service constraints	Lack of access to the Internet and telephone services. Issues with transportation to healthcare facilities	[36,38,39,40]
5	Social support resources	Risk of loneliness, sadness, and social disconnection during the pandemic	[35,36,37]
6	Mechanical ventilation and ambulatory care services	Lack of ambulatory care, higher need for intensive care units	[26,36,37,39,40]

### 3.9. Implications for Practice

The findings from this scoping review suggest a need for both universal and targeted healthcare support interventions to minimize the risk of excess dementia and COVID-19 morbidity among older African American adults. Universal healthcare support may include Medicaid expansion to enroll younger adults, reducing the risk of increased disease morbidity and mortality in older adults through preventive healthcare [50]. Targeted health interventions for older African Americans at risk of dementia and community-spread diseases such as COVID-19 may include the use of mobile outreach clinics to bring healthcare to their neighborhoods, increasing access. These interventions could result in sustainable healthcare access with community education about healthcare accessibility and resources, including the best use of Medicaid for which older African Americans are eligible. In some studies, it was shown that African Americans prioritized treatment and care that involved family and had less trust in health facilities [42,46]. This may be due to a long history of experiencing racial discrimination from healthcare personnel and neglect of their healthcare needs [27,44,49]. Taking proactive steps to ensure healthcare is available, approachable, and appropriate across color lines would go a long way toward bridging the avoidable healthcare accessibility gaps in which pandemics thrive and impact vulnerable patients such as older African American adults with dementia. This will require public health mandates such as universal healthcare. Additionally, a better understanding of related factors may become an interesting research field that will be necessary for formulating preventive strategies to curb healthcare accessibility and resource issues. 

### 3.10. Limitations of the Review and Suggestions for Further Research

There are several limitations in this scoping review. First, there have been very few studies published on healthcare accessibility and resources among older African Americans with dementia and COVID-19. Moreover, the studies have been mostly exploratory, as would be expected in a new area of research. Nonetheless, the findings are consistent with historical and contemporary trends in the healthcare participation of older African Americans, suggesting a need to frame future studies on socioeconomic theories that have proven to be effective in better understanding racialized healthcare in the United States.

## 4. Conclusions

The findings of this scoping review suggest that older African Americans with dementia and COVID-19 experience healthcare disparities due to the affordability of healthcare; the approachability, acceptability, and availability of healthcare access; and the inappropriateness of healthcare services. Medicaid resourcing to include elective treatments and brand medicines for older adults would benefit older African American adults with dementia and COVID-19. Older African American adults with dementia and COVID-19 may experience marginalization in terms of healthcare resources from financial insecurities and a lack of healthcare support, including a lack of information about the best care options regarding dementia and COVID-19. Healthcare accessibility and resource limitations lower the health-related quality of life of older African American adults with dementia and COVID-19. The emerging evidence shows that the structural inequities that prevail among older African American adults place them at risk of poor healthcare for dementia and COVID-19.

## Figures and Tables

**Table 1:** Literature review key topics and search terms.

	Key Concepts		Search Terms
	African American	OR	“Black Americans” OR “blacks”
AND	Older adults	OR	“Older adult” OR elderly OR geriatric OR aging OR senior OR “older people” OR “aged 65” OR 65 + OR elderly OR senior OR aged or older or elder or geriatric or “elderly people” OR “older people.”
AND	Dementia	OR	“Alzheimer” OR “cognitive impairment” OR “memory loss” OR “mild cognitive impairment” OR “age-associated cognitive impairment” OR “mild cognitive decline” OR “mild cognitive dysfunction”
AND	COVID-19	OR	“Coronavirus” OR “2019-ncov” OR “cov-19” OR “pandemic”
AND	Healthcare Accessibility	OR	“Health access” OR “access to healthcare” OR “health services accessibility”
AND	Resources	OR	“Support” OR “products” OR “services.”

**Table 2:** Summary of study participants and their characteristics of the included studies.

Studies	Type of Study	Database	Outcomes (Access/Health Resources)	Health Conditions	Characteristics
Ge et al., 2021 [35]	Exploratory study	Atla Religion Database with AtlaSerials	Social isolation affected by poor access to technology among older African American adults. Lack of culturally adapted treatments.	Dementia and COVID-19	N = 16 individuals with dementia and COVID-19 who were involved in single interviews over three months. All participants were Black or African American.
Gilstrap et al. 2022 [27]	Cross-sectional study	APA Psychinfo	Excess mortality among older adults with dementia, especially in Asian, Black, and Hispanic populations and people living in nursing homes, even in areas with low COVID-19 prevalence. Reduced healthcare access and resources (36.7%; 95% CI, 35.2–38.2% for older Black populations)	Dementia and COVID-19	N = 53,640,888 Among older adults diagnosed as having dementia in 2019, 63.5% were women, 2.7% were Asian, 9.2% were Black, 5.7% were Hispanic, 80.7% were White, and 1.7% were identified as other (included all races or ethnicities other than those given)
Holaday et al., 2022 [36]	Cross-sectional analysis	APA Psychinfo	Low odds of access to primary care (odds ratio = 0.72; 95 confidence interval (CI): 0.61–0.87; *p* < 0.001); high loneliness among older African American adults. Use of Medicare, financial insecurities, Internet/phone	Dementia and COVID-19	N = 11,114 community-dwelling Medicare beneficiaries and self-reported people with dementia and COVID-19
Kenerly et al., 2021 [26]	Empirical studies	Medline	In-hospital mortality, mechanical ventilation, reduced healthcare access, and resources among older African American adults. Intensive care unit shortages.	Dementia, COVID-19, heart disease, kidney disease, cerebrovascular disease, hypertension	N = 710 patients, 73 (10.3%) presented with altered mental status (AMS). They also presented with cardiovascular diseases, dementia, and COVID-19. The majority of the population were older African Americans (83.4%).
Kim et al., 2022 [37]	Longitudinal cohort study	Psychology and Behavioral Sciences	Primary outcomes are COVID-19 hospitalization. Severe outcomes are intensive care unit (ICU), intubation, dialysis, stroke, and in-hospital death. Reduced access to health resources for older African American adults. Lack of medications. Ambulatory care.	Dementia, COVID-19, stroke, intubation.	N = 47,219 cohort of older patients aged 65+ with laboratory-confirmed COVID-19 and dementia. Median age was 74 years; 47.4% were male, 24.3% non-Hispanic White, 23.3% non-Hispanic Black, and 18.4% Hispanic.
Sadler et al., 2020 [38]	Purposive sampling, open-ended qualitative interviews	Academic search complete	Reports on inability to access quality care and participate in medical appointments among older African American adults. Poor transportation. Financial insecurities. Telemedicine.	Dementia, COVID-19	N = 63 family caregivers interviewed for older adults with dementia and COVID-19 across eight states. About 89% of participants were female, 78% White, 10% Black, 5% Asian, 3% biracial, and 2% Native American. 36% of respondents reported that those they cared for received Medicaid
Silver et al., 2020 [39]	Empirical study (Retrospective study)	Academic search complete	Older African American adults were disproportionately represented in hospitalizations. Reduced healthcare accessibility (odds ratio = 0.92; 95% CI, 0.70–1.20) Lack of health education. Financial insecurities.	Dementia, COVID-19, respiratory problems, fever	N = 249 patients with dementia and COVID-19. The median age was 59; 44% were male, and 86% were aged ≥ 65 years or had ≥ 1 comorbidity. Overall, 87% were Black. Older African American adults had longer symptom duration at presentation (6.41 vs. 5.88 days; *p* = 0.05)
Wiese et al., 2021 [40]	Empirical study (dependent test design)	Medline	Limited access to healthcare, especially for rural-dwelling older African Americans. Lack of insurance. Financial insecurities. Cognitive screening.	Dementia, COVID-19	N = 60; 70% of the sample self-reported as older African American, Haitian Creole, or Hispanic with dementia and COVID-19; 75% were female.

## Data Availability

Not applicable.
